# Development of a novel patient reported outcome measure for health-related quality of life in amyotrophic lateral sclerosis (PROQuALS): study protocol

**DOI:** 10.1186/s12955-024-02286-3

**Published:** 2024-08-30

**Authors:** Jill Carlton, Philip Powell, Donna Rowen, Claire Williams, Alys Wyn Griffiths, Esther Hobson, Christopher McDermott

**Affiliations:** 1 Sheffield Centre of Health and Related Research (SCHARR), University of Sheffield, Sheffield, UK; 2 Division of Neuroscience, University of Sheffield, Sheffield, UK

**Keywords:** Amyotrophic lateral sclerosis (ALS), Discrete choice experiment (DCE), Health-related quality of life (HRQoL), Motor neuron disease (MND), Patient reported outcome measure (PROM), Preference-weighted measure (PWM), Quality adjusted life year (QALY)

## Abstract

**Background:**

Patient reported outcome measures (PROMs) can be used to assess the impact of health conditions upon an individual’s health-related quality of life (HRQoL). Whilst PROMs have been used to quantify the HRQoL impact of amyotrophic lateral sclerosis (ALS), existing instruments may not fully capture what matters to people living with ALS (plwALS) or be appropriate to be used directly to inform the cost-effectiveness of new treatments. This highlights a need for a new condition-specific PROM that can both capture what’s important to plwALS and be used in economic evaluation. This study has two key aims: 1) to produce a novel PROM for measuring HRQoL in plwALS (PROQuALS). 2) to value a set of items from the novel PROM to generate an associated preference-weighted measure (PWM) that will enable utility values to be generated.

**Methods:**

A mixed-methods study design will be conducted across three stages. Stage 1 involves concept elicitation and the generation of draft PROM content from a robust and comprehensive systematic review of HRQoL in ALS, with input from plwALS. Stage 2 consists of cognitive debriefing of the draft PROM content to ascertain its content validity (Stage 2a), followed by a psychometric survey (Stage 2b) to assess statistical performance. Evidence from Stage 2 will be used to make decisions on the final content and format of the novel PROM. Stage 3 will involve valuation and econometric modeling using health economics methods to generate preference weights, so a PWM derived from the novel PROM can be used in the cost-effectiveness analyses of treatments. Patient and clinical advisory groups will have critical, collaborative input throughout the project.

**Discussion:**

The novel PROM will be designed to comprehensively assess important aspects of HRQoL to plwALS and to quantify HRQoL in terms of subjective impact. The PROQuALS measure will be available for use in research and healthcare settings. The associated PWM component will extend and enable the use of PROQuALS in cost-effective analyses of new treatments for ALS.

**Trial registration:**

Not applicable.

## Background

Amyotrophic lateral sclerosis (ALS) is a life-shortening, rare neurodegenerative condition that has a significant burden on patients’ lives [1]. The disease causes progressive deterioration in nerve cells responsible for muscle movement and is eventually fatal, with an estimated median survival of 2.4 years post-diagnosis [2]. Incidence of ALS has been estimated recently at 1.59 per 100,000 person-years globally, with increased incidence by age (up until ~ 70 years), and a trend of increasing incidence over time [3]. Presently, there is no cure for ALS, so clinical management is based on slowing progression and maximizing patients’ health-related quality of life (HRQoL). Health-related quality of life is a multidimensional construct that transcends physical health indicators and includes a person’s “physical, psychological, and social functioning associated with an illness or its treatment” [4]. Increasingly, HRQoL data forms an important outcome in clinical trials, and, in specialized forms, is used to generate quality-adjusted life years (QALYs) that feature in cost-effectiveness analyses of healthcare interventions through health technology assessments [5]. The concept of HRQoL is inherently subjective and is most frequently assessed using questionnaires known as patient reported outcome measures (PROMs), which are completed by patients themselves, or, if that is not possible, by proxy respondents. Patient reported outcomes vary in content and can be ‘generic’ (i.e., designed to be applicable across health conditions and maximize comparability) or ‘condition-specific’ (i.e., designed to be sensitive to specific health conditions and maximize content validity) [6].

Many PROMs exist to assess (aspects of) HRQoL and over 111 have been used in people living with ALS (plwALS) [7]. However, the choice of PROMs used to gauge HRQoL in ALS (and other health conditions) is inconsistent and varies across studies. There are also critical limitations to existing PROMs used to assess HRQoL in ALS. First, there are concerns over the comprehensiveness of existing PROMs to assess all aspects of HRQoL that matter to plwALS, with a dominant focus on physical functioning (rather than social or psychological functioning) [7]. Second, existing condition-specific HRQoL PROMs in ALS typically use a frequency and/or agreement response scale to assess the degree that different aspects of HRQoL are affected, but they do not assess how much this degree of impact matters to plwALS. It is possible to experience something a lot, but acknowledge that it is not important to you, or vice versa. Third, many existing condition-specific PROMs used to assess HRQoL in ALS are not preference-weighted, which means the data from them cannot be used to calculate QALYs for use in cost-effectiveness analyses of interventions for ALS. Furthermore, many existing (condition-specific) HRQoL PROMs in ALS are not suitable for adaptation for use for economic valuation because they were not developed with that criterion in mind (c.f., Peasgood et al., 2021 [8]).

The objective of this study is to develop a novel PROM (Patient Reported Outcome measure of Quality of life in Amyotrophic Lateral Sclerosis [PROQuALS]) to assess HRQoL in ALS that: (i) comprehensively captures aspects of HRQoL that matter to plwALS; (ii) uses a response scale that captures the subjective impact of ALS on different aspects of HRQoL on the person completing the PROM; and (iii) is amenable to economic evaluation to derive an associated preference-weighted measure (PWM). The work proceeds through three stages, detailed in this protocol. Stage 1 involves concept elicitation and the generation of draft PROM content from a robust and comprehensive systematic review of HRQoL in ALS [7], with input from plwALS. Stage 2 consists of cognitive debriefing of the draft PROM content to ascertain its content validity (Stage 2a), followed by a psychometric survey (Stage 2b) to assess statistical performance. Evidence from Stage 2 will be used to make decisions on the final content and format of the novel PROM.

The refined PROM generated and finalised in Stage 2 cannot be used to directly inform cost-effectiveness analyses and therefore an ALS-specific PWM will be generated for this purpose. A PWM consists of a) a classification system comprised of dimensions of HRQoL and associated severity levels that can be used to categorise the HRQoL of all patients, and b) a scoring system derived from preferences which enable a utility value to be generated for every state defined by the classification system. Stage 3 will involve valuation and econometric modeling using health economics methods to generate preference weights, so a PWM derived from the novel PROM can be used in the cost-effectiveness analyses of treatments. Both patient and clinical advisory groups have been established and will have critical, collaborative input throughout the project.

## Methods

### Project governance (including patient involvement)

**Fig. 1:**
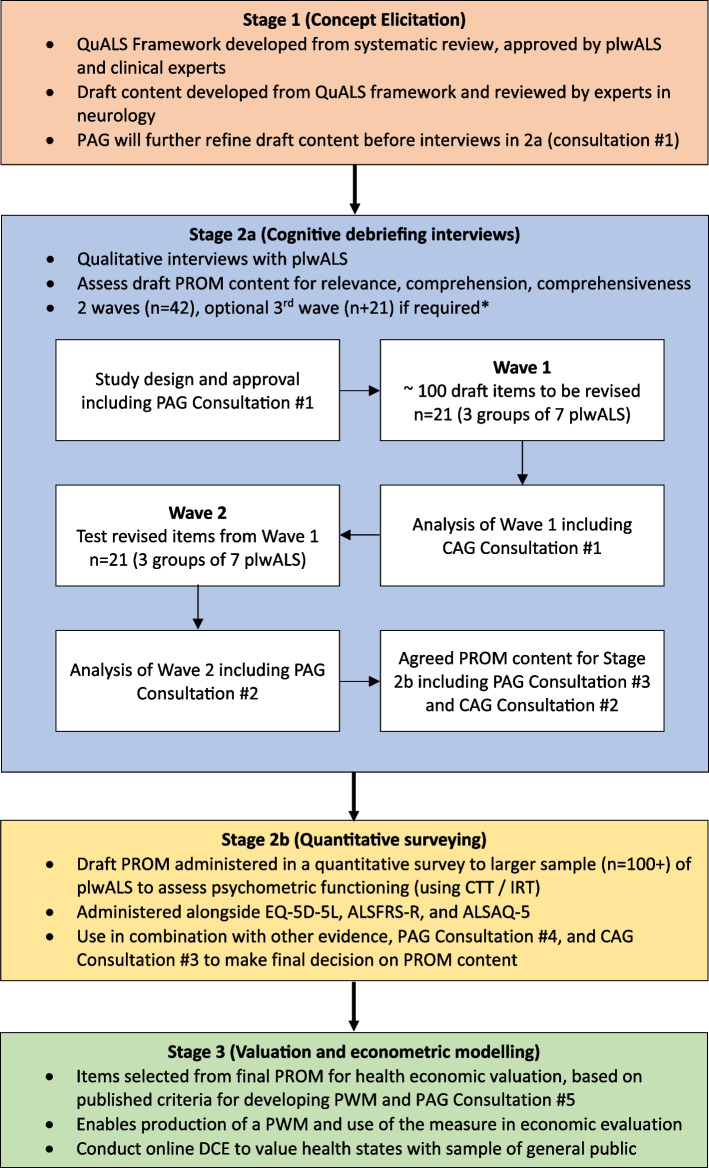
Overview of project. ALSAQ-5 = Amyotrophic Lateral Sclerosis Assessment Questionnaire; ALSFRS-R = Revised Amyotrophic Lateral Sclerosis Functional Rating Scale; CAG = Clinical Advisory Group; CTT = Classical test theory; DCE = Discrete choice experiment; IRT = Item response theory; PAG = Patient Advisory Group; PlwALS = People living with ALS; PROQuALS = Patient Reported Outcome measure of Quality of life in Amyotrophic Lateral Sclerosis; PROM = Patient reported outcome measure; PWM = Preference-weighted measure; QuALS = Health-related quality of life in amyotrophic lateral sclerosis

The research has three governance groups who will be actively involved at key stages: the research team (comprising a core group of researchers including PROM developers, health economists, and clinical academics); the Clinical Advisory Group (CAG) (comprising a diverse group of clinicians and clinical academics who specialise in ALS); and the Patient Advisory Group (PAG) (comprising adults with ALS). The CAG and PAG will collaborate with the research team at critical times throughout the research project (Fig. 1), informed by an existing framework designed to ensure lived experience is integrated fully into PROM development [9]. Representatives of the PAG were first engaged in the research via input into the conceptual framework underlying item generation in Stage 1 [7]. While the methods/study design are already defined, the PAG (and CAG) will continue to have input into the development of PROQuALS throughout the project, including in content decisions, interpreting results, and helping to select which items are taken forward for preference weighting.

### Project aims

This project has three aims:

1. To generate draft PROM content for measuring HRQoL in plwALS based on a comprehensive literature review and conceptual framework of HRQoL in ALS (QuALS).
2. To produce a finalized novel PROM for measuring HRQoL in plwALS, utilising qualitative methods (cognitive debriefing interviews), quantitative methods (psychometric analyses), and expert input from collaborators (PAG and CAG).
3. To value a set of items from the novel PROM to generate an associated preference-weighted measure (PWM) that will enable utility values to be generated in order to derive QALYs for use in cost-effectiveness analyses.

The research is split into three iterative stages that map onto the aims above (Fig. 1).

#### Stage 1: concept elicitation

The purpose of Stage 1 is to generate draft content for the novel PROM. The underlying conceptual framework for the PROQuALS instrument is the QuALS framework. QuALS was developed following a systematic review of the literature on HRQoL in ALS, and was ratified by plwALS and clinical experts [7]. The QuALS framework was used to inform the development of draft PROM content (including items, instructions, and response options) for the PROQuALS. A total of 101 draft items, across seven QuALS domains (Activities; Physical Health; Autonomy; Cognition; Feelings and Emotions; Self-identity; and Relationships) have been drafted by the core research team. Draft instructions were developed based on a structure that has been received well in other PROMs developed by the research team [10, 11]. The draft PROM features a 2-week recall period to balance issues of having a sufficient time period to capture different aspects of HRQoL with issues of recall. Two weeks is also the modal recall period used in ALS-specific PROMs assessing aspects of HRQoL [7]. An initial draft 4-point response scale that assesses subjective impact of each aspect of HRQoL (i.e., “how much of a problem for you was…”) was developed for testing and differentiates this PROM from other condition-specific measures in ALS. The draft PROM content will be further refined through collaboration with the PAG prior to Stage 2a (cognitive interviews).

#### Stage 2: refining the descriptive system

The purpose of Stage 2 is to revise the draft content developed in Stage 1 to produce a final HRQoL PROM for use in ALS. Stage 2 has two substages (Fig. 1).

##### Stage 2a – cognitive debriefing interviews

Draft PROM content will be assessed through qualitative methodology (cognitive debriefing interviews). The purpose of the interviews is to determine the content validity of the draft PROM content (i.e., to assess its relevance, comprehensiveness, and comprehensibility [12]). This includes the instructions, items, response options, and recall period. Interviews will be informed by a topic guide based on published guidance from The Professional Society for Health Economics and Outcomes Research (ISPOR) and COnsensus-based Standards for the selection of health Measurement INstruments (COSMIN) [13, 14]. Interviews will be conducted online to facilitate inclusivity, and transcribed intelligent verbatim. Due to the number of draft items generated in Stage 1, and reflecting upon response burden, interviews will be conducted over a minimum of two iterative waves. Each participant will be asked to comment upon a maximum of 40 items only (incorporating full sets of items from each HRQoL theme). The methodology outlined, including sample size requirements, are based on COSMIN guidance [15]. A minimum number of 42 interviews will be conducted in Stage 2a. Potential participants will be recruited by advertising through relevant charitable organisations based in the United Kingdom (UK) (e.g., MND Association). Adaptations will be made to allow for participation from individuals whose speech is impaired. This includes (but is not limited to) the use of augmentative communication devices and/or ‘chat’ function in the online interview platform. Purposive sampling will be used, based on a predefined target sampling framework to ensure representation across age, gender, and years since diagnosis of ALS (Table 1).

**Table 1:** Target sampling framework for each wave of the cognitive interviewing

Group	Age			Gender		Years since diagnosis			Total
	18–40	41–65	66 +	Men	Women	1–2	3–4	4 +	
1	1	3	3	4	3	2	4	1	7
2	1	3	3	4	3	2	4	1	7
3	1	3	3	4	3	2	4	1	7

Group 1 will be shown items covering the themes ‘Physical health’ and ‘Activity’; Group 2 ‘Relationships’, ‘Self-identity’, and ‘Cognition’; Group 3 ‘Autonomy’ and ‘Feelings and Emotions’

The qualitative data will be used to assess each component of the draft PROM, including items, response options, and/or completion instructions against existing content validity criteria for relevance, comprehensiveness within themes(s), and comprehensibility. Analysis will be conducted wherever possible alongside data collection to best reciprocally inform each process. Analysis will follow best practice guidance [15], with 20% of transcripts dual-coded. Wave 2 will encompass the same approach. Following Wave 1, interview results will be discussed with the CAG to help ratify any decisions of modification to the PROM as a result of cognitive debriefing. Following Wave 2, the interview results will be discussed with the PAG in the same manner. If necessary, a further (and final) wave of interviews will be conducted to test any final modifications to the PROM content. At the end of Stage 2a, proposed PROM content is to be taken forward to Stage 2b following consultation with both the CAG and PAG (Fig. 1).

##### Stage 2b – Quantitative surveying

The refined PROM content will be included in a quantitative survey online alongside a selection of existing validated PROMs, including a generic PWM (EQ-5D-5L [16]), a condition-specific measure of disease progression (ALSFRS-R [17]), and a condition-specific measure of health status, Amyotrophic Lateral Sclerosis Assessment Questionnaire (ALSAQ-5 [18]). These PROMs were selected based on frequency of use in the field and to minimize participant response burden (alongside the draft PROM and other questions in the survey). The inclusion of additional PROMs allows for the psychometric performance of the draft PROM to be assessed (i.e., its construct validity).

The survey will be hosted online on Qualtrics, with opportunity sampling of plwALS via relevant charitable organizations based in the UK (e.g., MND Association). As well as self-reported (by plwALS) subjective measures of health, basic clinical (to determine King’s staging [19] and type of ALS) and sociodemographic data will be collected to enable descriptions of the sample and for additional psychometric analyses (e.g., construct validity via predicted relationships and known-groups analyses against underlying characteristics). Whilst prior PROM development studies have used samples of > 300 participants [10, 20], it is important to reflect that ALS is a rare condition and adaptations need to be made. Other studies have demonstrated that smaller sample sizes are appropriate when developing PROMs in rare conditions [11]. We will adopt a pragmatic approach and set a minimum sample size of 100 participants. Recruitment for the survey will be open for at least 4 months and until the minimum sample size has been achieved.

Prior to analysis, data will be cleaned and a priori data quality checks will be performed, similar to those outlined in Carlton et al., 2024 [10]. Analysis will be conducted in R, with descriptive analysis of item responses including item distributions, floor/ceiling effects, and missing data values explored. Classical test theory (CTT) and item response theory (IRT) approaches will be undertaken, with analyses adapted for the resultant sample size. Assuming a sufficient sample size, a combination of confirmatory factor analysis (CFA) and exploratory factor analysis (EFA) will be used to understand the dimensionality of the scale. Items will also be considered for potential redundancy based on the strength of their correlation with other items. Following any necessary revisions to the scale through CTT, and again assuming a sufficient sample size, IRT on unidimensional scales will be used to assess individual item performance (in terms of item fit, ordering of thresholds, and differential item functioning). Assumptions of IRT (i.e., unidimensionality, monotonicity, and local independence) will be assessed prior to use.

All decisions on final item selection for the PROM will incorporate and balance evidence from Stage 2a (qualitative interviews) and Stage 2b (psychometric survey). Trade-offs may have to be made between achieving sufficient content validity (particularly comprehensiveness) of the measure by including content that is important to plwALS and achieving optimal psychometric performance. All decisions on final item selection will be made consultatively within the research team, PAG, and CAG, and documented via an ‘item tracking matrix’.

Once the final PROM is confirmed, exploratory analysis will be undertaken to assess the PROM’s internal consistency reliability (using Cronbach’s alpha) and construct validity. The reliability and validity of subdomain scores (e.g., physical, psychological, social functioning) will be explored where possible. We expect scores on the PROQuALS to correlate significantly (i.e., at > 0.3) with scores on the ALSFRS-R, ALSAQ-5, and EQ-5D-5L, in decreasing order of magnitude. We will explore known-group validity by investigating how condition severity (as determined by King’s Staging) affects HRQoL (including subdomain scores). We expect disease severity to be negatively associated with PROQuALS scores, with a stronger relationship for physical functioning domain score. The content validity of the final PROM will be re-assessed through cognitive interviews with seven plwALS, following similar sampling and methodological procedures to that described in Stage 2a.

#### Stage 3: Valuation and econometric modelling to generate an ALS-specific preference-weighted measure

The refined PROM generated and finalised in stage 2 cannot be used to directly inform cost- effectiveness analyses and therefore an ALS-specific PWM is generated for this purpose. A PWM consists of a) a classification system comprised of dimensions of HRQoL and associated severity levels that can be used to categorise the HRQoL of all patients, and b) a scoring system derived from preferences which enable a utility value to be generated for every state defined by the classification system.

##### Stage 3.1 Deriving a classification system from the PROM developed in Stage 2

The PROM developed in Stage 2 will potentially contain multiple items reflecting the same or similar underlying constructs within each domain and is expected to contain over 10 items. A PWM requires a parsimonious number of items since a larger number of items with multiple items tapping identical constructs are not feasible to value in Stage 3.2. Psychometric analyses will be conducted using the quantitative data generated in Stage 2b, and published criteria for developing PWM [8] will be applied, and results will be considered by the research team, consisting of PROM developers, health economists and clinical professionals. The PAG will also be consulted and collaborate in selecting items for the PWM. This will enable selection of the best-performing items most appropriate for valuation that reflect the underlying constructs and dimensions of the PROM deemed important to patients in Stage 2a.

##### Stage 3.2 Eliciting and modelling preferences to generate utility values

An online discrete choice experiment (DCE) will be conducted to elicit preferences for the states defined by the classification system. DCEs are commonly used for this purpose to enable the generation of utility values for PWMs and condition-specific PWMs in particular [21]. Due to the large number of states defined by the classification system, it is infeasible to include all and hence the DCE tasks will be selected statistically taking into account the modelling that will be undertaken on the DCE data. Participants will be representative of the UK general public in accordance with NICE recommendations for utility values to generate QALYs for cost-effectiveness analyses [22]. Participants will be recruited from existing panels of participants willing to answer surveys by a market research company. The sample (N = 1000) will be with participants of the UK general population representative for age and gender. The DCE results will be modelled using regression analyses to generate a utility value for every health state that can be directly used to inform cost-effectiveness analyses.

## Discussion

The research follows best practice guidance for the development of new instruments [14, 23, 24], however due to the rare nature of the condition, a pragmatic approach has been taken where appropriate. Whilst every effort has been made to ensure inclusivity within the project, it is not possible to address all areas. The PROQuALS questionnaire will be developed in the UK, and efforts will be made to facilitate participation across different ethnicities. Due to resource limitations, an international approach to recruitment is not possible, however the international nature of the CAG will help to mitigate any significant cultural implications. Further work will be required to develop and validate other language versions of the PROQuALS, and to determine whether any cultural adaptations of the English (UK) version are needed. As with the development of all PROMs, further studies will be required to fully assess the psychometric properties of the final PROQuALS. The PWM will be developed based on general public preferences as advocated by NICE [22]. These preferences may differ to those with lived experience, and we would advocate that further work is conducted to explore this.

Collaborative engagement with plwALS and health care professionals will help ensure the new PROM will be useful in both research and clinical contexts. Engagement will follow existing frameworks will be reported using recognised best practices [9, 25]. Purposive sampling in the cognitive interviewing stage will be used to ensure views of plwALS for different durations, and thus likely different degrees of disease severity, are represented. However, it is possible that, particularly in the quantitative surveying stage, the views of those living with more severe disabilities and who are newly diagnosed may be underrepresented. The voices and input of the PAG and CAG will be drawn upon to help mitigate this concern.

The PROQuALS instrument will be designed to capture the impact ALS has on a person’s HRQoL. There is a balance between creating a questionnaire that is fully comprehensive (i.e., includes all areas that ALS impacts upon an individual) but also acceptable in terms of the number of questions within it. It is recognised that respondent burden can compromise data quality from PROMs [26]. Furthermore, parsimony is an important characteristic of a PROM and is linked to satisfactory psychometric performance. Thus, trade-offs often need to be made in practice when balancing these different aspects in PROM design. Accordingly, our focus will be on ensuring ‘core comprehensiveness’, that all aspects of HRQoL of a sufficient level of importance to plwALS will be included in the PROM. All decisions will be fully documented and made in conjunction with the PAG and CAG working on the project.

In summary, the proposed research will produce a novel PROM (Patient Reported Outcome measure of Quality of life in Amyotrophic Lateral Sclerosis [PROQuALS]) and associated PWM to assess HRQoL in ALS. The novel PROM will be designed to comprehensively assess important aspects of HRQoL to plwALS and to quantify HRQoL in terms of subjective impact. The associated PWM component will extend and enable the use of PROQuALS in cost-effective analyses of new treatments for ALS.

## Data Availability

The primary output of this research will be a condition-specific PROM for assessing HRQoL in plwALS, and associated value set for use in cost-effectiveness evaluations. The results of the project will be disseminated via at least two journal manuscripts (i.e., development of the PROQuALS, and deriving utility weights). All manuscripts will be published open access, with accompanying anonymised data uploaded to an online repository. Results will also be shared at national and international conferences. Non-technical reports of the findings, including interim findings, will be co-produced with the PAG and will be disseminated widely.

## Abbreviations

ALS: Amyotrophic lateral sclerosis
CAG: Clinical Advisory Group
CTT: Classical test theory
DCE: Discrete choice experiment
HRQoL: Health-related quality of life
IRT: Item response theory
PAG: Patient Advisory Group
PlwALS: People living with ALS
PROQuALS: Patient Reported Outcome measure of Quality of life in Amyotrophic Lateral Sclerosis
PROM: Patient reported outcome measure
PWM: Preference-weighted measure
QALY: Quality adjusted life year
QuALS: Health-related quality of life in amyotrophic lateral sclerosis

## Data availability

No datasets were generated or analysed during the current study.
